# Synchronization and Inter-Layer Interactions of Noise-Driven Neural Networks

**DOI:** 10.3389/fncom.2017.00002

**Published:** 2017-01-31

**Authors:** Anis Yuniati, Te-Lun Mai, Chi-Ming Chen

**Affiliations:** Department of Physics, National Taiwan Normal UniversityTaipei, Taiwan

**Keywords:** biological neural networks, inter-layer interactions, noise-driven synchronization, spike-timing-dependent plasticity, synchronous firing, computer simulation, developing neural networks, repair mechanism of neural networks

## Abstract

In this study, we used the Hodgkin-Huxley (HH) model of neurons to investigate the phase diagram of a developing single-layer neural network and that of a network consisting of two weakly coupled neural layers. These networks are noise driven and learn through the spike-timing-dependent plasticity (STDP) or the inverse STDP rules. We described how these networks transited from a non-synchronous background activity state (BAS) to a synchronous firing state (SFS) by varying the network connectivity and the learning efficacy. In particular, we studied the interaction between a SFS layer and a BAS layer, and investigated how synchronous firing dynamics was induced in the BAS layer. We further investigated the effect of the inter-layer interaction on a BAS to SFS repair mechanism by considering three types of neuron positioning (random, grid, and lognormal distributions) and two types of inter-layer connections (random and preferential connections). Among these scenarios, we concluded that the repair mechanism has the largest effect for a network with the lognormal neuron positioning and the preferential inter-layer connections.

## Introduction

Many sensory systems of animals, such as insects, frogs, and primates, have shown synchronized and periodic neural activities in the early stages. Experiments on lower mammals have demonstrated that activity-driven synchronization of neurons may occur during development and learning (Markram et al., 1997; Ben-Ari, 2001). For example, immature pyramidal neurons of the rat hippocampus start to receive sequentially established synaptic inputs around birth (Tyzio et al., 1999) and the hippocampal network generates periodic synchronized firings during the first two postnatal weeks (Ben-Ari et al., 1989). Such periodic and synchronized firing of large number of neurons can lead to network oscillations that have been observed in many brain systems, such as hippocampus (Fisahn et al., 1998; Csicsvari et al., 2003), prefrontal cortex (van Aerde et al., 2008), and visual cortex (Gray et al., 1992). It is believed that oscillations are relevant in various cognitive functions, such as learning (Miltner et al., 1999), attention (Fries et al., 2001), temporal binding (Engel et al., 2001), working memory (Haenschel et al., 2009), and memory consolidation (Axmacher et al., 2006).

Synchronous oscillatory activities are relevant for the development of cortical circuits, as demonstrated by the involvement of neural synchrony in synaptic plasticity and changes in the synchronization frequency of neural oscillations during development. From invasive electrophysiology in non-human primates and electro- and magnetoencephalographic (EEG/MEG) recording in humans, there is growing evidence suggesting that synchronous oscillatory activities are responsible for various cognitive and perceptual functions (Auerbach et al., 2001; Buzsaki, 2002; Buzsáki and Draguhn, 2004; Buzsáki, 2005; Kahana, 2006; Wang, 2010). In particular, precise synchronization of distributed neural responses is established by neural rhythms in the beta/gamma range (20–100 Hz), which play an important role in linking synchronized oscillations and cortical computations. Further experiments also suggest that the long-distance coordination of gamma-oscillations is related to alpha activity (8–12 Hz) and large-scale integration of subsystems for the formation and recall of memories are supported by theta activity (4–8 Hz). Generally, the synchronization frequency correlates with the distance over which synchronization occurs, i.e., short distance synchronization occurs at higher frequencies while long distance synchronization occurs at lower frequencies. Although theta activities are often driven by septal and entorhinal inputs, it is believed that gamma oscillations are intrinsically generated (Penttonen et al., 1998; Bartos et al., 2001).

It has been proposed that brain cortical function is mediated by dynamic modulation of coherent firing in groups of neurons (Vaadia et al., 1995; Fujii et al., 1996; Breakspear et al., 2004). The physiological data from the monkey prefrontal and visual cortices support the concept of dynamical cell assemblies that may spontaneously organize themselves temporarily by correlated firing of their spiking activity in response to external events. Based on this hypothesis, our understanding of the brain's processing and integration of information is that neural assemblies, composed of networks of neurons, are the basic computational units, which sporadically share information, and transiently use dynamical connections. By associating logical states to neuronal synchronous dynamics, it has been shown that the usual Boolean logics can be recovered and a universal Turing machine can be constructed (Zanin et al., 2011). The inter-network interactions could contribute to the variety of oscillation patterns and have been systematically investigated using two model networks with distinct oscillation frequencies (Avella Gonzalez et al., 2014). Despite the enormous efforts that have been devoted to the understanding of the brain's functions, its picture is still far from being complete.

The human brain is one of the most complicated neural networks, consisting of one hundred billion neurons and five quadrillion synaptic connections. Recent advances in multi-neuronal recording methods have discovered that groups of neurons can form physiological units and generate emergent functional properties, which are not *a priori* predictable based on the properties of individual neurons but arise from interactions among neurons in a neural network. As a new paradigm for neuroscience, modeling neural networks has the potential to bridge the gap in our knowledge about neurons and the whole brain. At the moment, it is more feasible to computationally study the dynamics of simple neural networks than the whole-brain dynamics. Our previous studies have shown that the intrinsic noise-driven dynamics could lead to network synchronization at the frequency range of gamma oscillations for culture samples of neural networks prepared from the cerebral cortex of embryonic rats (Jia et al., 2004; Chao and Chen, 2005; Lin et al., 2011). In studying the noise-driven synchronization dynamics of a developing neural network consisting of 50 neurons (Jia et al., 2004; Lin et al., 2011), initially there were no connections between neurons in the network. As the network developed with culturing time, intra-layer connections were established between neurons. Our investigation showed a logarithmical relationship between the synchronous firing frequency and the culturing time of the network by using the Hodgkin-Huxley (HH) (Hodgkin and Huxley, 1952) neuron model and two types of learning rules. This observation is consistent with the data from experiments on growing cultural neural networks prepared from embryonic rats (Jia et al., 2004). In this computational study, we consider similar simple systems for computer simulations and investigate the fundamental mechanism of the synchronized oscillations in coupled neural networks. We aim to explore the intrinsic dynamic behaviors of a neural network consisting of two coupled neural layers in a noisy environment by considering different forms of synaptic plasticity and network structures. Our network model and method of simulation are described in Section Methods. In Section Results and Discussion, we first study the phase diagram of a developing neural network (a single layer of 50 neurons) and discuss possible phases in the network activities. Inter-layer synaptic connections are then introduced to two independent neural layers to investigate their interactions. We discuss a possible repair mechanism for neural networks, which can set a non-synchronous layer off firing synchronously by its momentary coupling to a synchronous layer. The efficiency of this repair mechanism is analyzed for three types of neuron positioning on each layer and two types of inter-layer connections. We conclude our study in Section Conclusion.

## Methods

In this section, we presented our neural network model and numerical method for investigating the interaction between two coupled neural layers, each consisting of 50 neurons. The activities of neurons in a neural network were modeled based on the Hodgkin-Huxley (HH) neuron model described in Section The Neuron Model. For the activity-dependent development of neural networks, both the spike-timing-dependent plasticity (STDP) and the inverse STDP were considered for the learning of synapses (Hopfield and Brody, 2004), as discussed in Section Synaptic Plasticity. In the absence of an external input, in Section Neural Networks in a Noisy Environment, we modeled synaptic noise in a noisy neural network to study the intrinsic dynamic behaviors of the network. In Section Simulating Coupled Neural Networks, we explicitly constructed various coupled neural networks by considering possible types of neuron distribution on each layer as well as possible mechanisms for intra- and inter-layer synaptic connections. We note that the neuron model, numerical method, and parametric values in Sections The Neuron Model–Neural Networks in a Noisy Environment are similar to those in our previous study (Lin et al., 2011), which produced results that are consistent with experimental findings (Jia et al., 2004).

### The neuron model

In 1952, Hodgkin and Huxley developed a mathematical model to explain the ionic mechanisms underlying the initiation and propagation of action potentials (APs) in the squid giant axon (Hodgkin and Huxley, 1952). In the HH model, each component of an excitable cell is treated as an electrical element. The lipid bilayer is represented as a capacitance (*C*_*m*_). Voltage-gated ion channels and leak channels are represented by electrical conductance (*g*_Na_, *g*_K_, and *g*_L_ denote the maximum conductance per surface area of the sodium, potassium and leak currents). Finally, ion pumps are represented by current sources (*I*). Explicitly, the dynamics of neurons is described by the following equations:

(1) $$\begin{array}{l} C_{m}\frac{dV_{i}}{dt}=g_{\text{Na}}m_{i}^{3}h_{i}(V_{\text{N}a}-V_{i})+g_{\text{K}}n_{i}^{4}(V_{\text{K}}-V_{i})\\ \;+\;g_{\text{L}}(V_{\text{rest}}-V_{i})+I_{i}^{\text{syn}}(t), \end{array}$$

(2) $$\begin{array}{l} \frac{dm_{i}}{dt}=(1-m_{i})\frac{25-V_{i}}{10\left[\exp\;\left(\frac{25-V_{i}}{10}\right)-1\right]}\\ \;-\;m_{i}\left[4\;\cdot \;\exp\;\left(\frac{-V_{i}}{18}\right)\right], \end{array}$$

(3) $$\begin{array}{l} \frac{dn_{i}}{dt}=(1-n_{i})\;\cdot \;\frac{1-0.1\;\cdot \;V_{i}}{10\;\left[\exp\;\left(\frac{10-V_{i}}{10}\right)-1\right]}\\ \;-\;n_{i}\left[0.125\;\cdot \;\exp\;\left(\frac{-V_{i}}{80}\right)\right], \end{array}$$

(4) $$\begin{array}{l} \frac{dh_{i}}{dt}=(1-h_{i})\;\cdot \;0.07\;\cdot \;\exp\;\left(\frac{-V_{i}}{20}\right)\\ \;-\;\frac{h_{i}}{\exp\;\left(\frac{30-V_{i}}{10}\right)+1}, \end{array}$$

where a set of four time-dependent variables (*V*_*i*_, *m*_*i*_, *n*_*i*_, *h*_*i*_) were used to describe the activity of *i*-th neuron. Here *V*_*i*_ is the membrane potential, *m*_*i*_ and *h*_*i*_ are the activation and inactivation variables of the sodium current, and *n*_*i*_ is the activation variable of the potassium current. *V*_Na_, *V*_k_ and *V*_rest_ are the corresponding reversal potentials. Typical values of the parameters were chosen as *C*_*m*_ = 1.0 μF/cm^2^, *g*_Na_ = 120.0 mS/cm^2^, *g*_K_ = 36.0 mS/cm^2^, *g*_L_ = 0.3 mS/cm^2^, *V*_Na_ = 115.0 mV, *V*_k_ = −12.0 mV, and *V*_rest_ = 10.6 mV. $I_{i}^{\text{syn}}\left(t\right)$ is the total synaptic current, a sum of output currents, $I_{j}^{\text{out}}\left(t-T_{0}\right)$, from connected neurons in the network with a synaptic strength *w*_*ij*_ and a signal delay time *T*_0_. Explicitly, the total synaptic current is expressed as $I_{i}^{\text{syn}}\left(t\right)=\underset{j}{\sum }w_{ij}\cdot I_{j}^{\text{out}}\left(t-T_{0}\right)$. For simplicity we approximated the output current *I*_*j*_^out^(*t*) as a step function with a duration 0.1 ms and an amplitude $I_{\max}\cdot {\left\{1+\exp\left[-0.002\cdot V_{j}^{\text{peak}}\left(t\right)\right]\right\}}^{-1}$, where *V*_*j*_^peak^(*t*) is the peak value of the AP of *j*-th neuron at time *t* and *I*_max_ is the maximum output current from a neuron (Koch, 1999). Typical values of *I*_max_ and *T*_0_ used in our simulations are 25 nA/cm^2^ and 9 ms, respectively. The strength or efficacy (*w*_*ij*_) of synaptic transmission at preexisting synapses is subject to activity-dependent modification, and will be discussed in detail in Section Synaptic Plasticity.

For a small enough time step Δ*t*, the neuronal membrane potential *V*(*t*) in Equation (1) can be solved numerically using the Euler method as:

(5) $$\begin{array}{l} V_{i}(t+\Delta t)=V_{i}(t)+\frac{\Delta t}{C_{m}}\text{ ​}\{I_{i}^{syn}(t)+g_{\text{Na}}m_{i}{\left(t\right)}^{3}h_{i}(t)[V_{\text{N}a}-V_{i}(t)]\\ \;+\;g_{\text{K}}n_{i}{\left(t\right)}^{4}[V_{K}-V_{i}(t)]+g_{\text{L}}[V_{\text{rest}}-V_{i}(t)]\}, \end{array}$$

where *n*(*t*), *m*(*t*), and *h*(*t*) are obtained from solving Equations (2) to (4) (Koch, 1999). For a brief derivation, Equations (2)–(4) can be expressed as $\frac{dz}{dt}=\alpha_{z}\left(V\right)\left(1-z\right)-\beta_{z}\left(V\right)z\;=\;\left(z_{\infty }-z\right)/\tau_{z}$, where *z*_∞_ = α_*z*_/(α_*z*_+β_*z*_), τ_*z*_ = 1/(α_*z*_+β_z_), α_*z*_ and β_*z*_ are voltage-dependent rate constants in Equations (2)–(4), and *z* represents *m, n*, or *h*. When the membrane potential is held at a constant value (such as by voltage clamp), the solution of the three gating equations can be obtained as *z*(*t*) = *z*_∞_ + (*z*_0_ − *z*_∞_) exp (−*t*/τ_*z*_), where *z*_0_ = 0.05, 0.32, and 0.60 respectively for *z* = *m, n*, and *h*. In general, the neuronal membrane potential obtained from Equation (5) is more accurate with a smaller step size Δ*t*. In our experience, Δ*t* = 0.01 ms will be good choices for the size of time step. In this study, for the efficiency of the network simulations and the accuracy of simulation results, we chose Δ*t* = 0.01 ms.

### Synaptic plasticity

In neural systems, a synapse between two neurons can change its strength in response to either use or disuse of transmission over synaptic pathways (Hughes, 1958). Previously, the Hebbian learning rule was suggested that the synaptic strength could increase if the presynaptic neuron repeatedly and persistently stimulates the postsynaptic neuron to generate APs. More recent experiments have observed a spike-timing-dependent synaptic plasticity (STDP): repeated presynaptic spike arrival a few milliseconds before postsynaptic action potentials leads in many synapse types to long-term potentiation (LTP) of the synapses, whereas repeated spike arrival after postsynaptic spikes leads to long-term depression (LTD) of the same synapse. Previous experiments also demonstrated that postsynaptic APs are initiated in the axon and then propagate back into the dendritic arbor of neocortical pyramidal neurons, evoking an activity dependent dendritic Ca^2+^ influx that could be a signal to induce modifications at the dendritic synapses that were active around the time of AP initiation (Markram et al., 1997). Therefore, the synaptic efficacy can be regulated depending on the precise timing of postsynaptic APs relative to excitatory postsynaptic potentials. The characteristic time intervals for synaptic modifications are found to be 17 ms for facilitation and 34 ms for depression for layer 5 pyramidal neurons in somatosensory cortex (Bi and Poo, 2001). Such a STDP rule was introduced in our simulations by considering a change in the synaptic strength (Δ*w*_*ij*_) due to learning at each time step as Bi and Poo (2001):

(6) $$\begin{array}{c} \Delta w_{ij}(\Delta \tau )=\{\begin{array}{c} \begin{array}{cc} A_{+}\exp\;\left(-\Delta \tau /\tau_{+}\right) & \Delta \tau >0 \end{array}\\ \begin{array}{cc} -A_{-}\exp\;\left(\Delta \tau /\tau_{-}\right) & \Delta \tau <0 \end{array} \end{array}, \end{array}$$

where Δτ is the time of the postsynaptic spike minus the time of the presynaptic spike. The parameters τ_+_ and τ_−_ determine the ranges of pre-to-postsynaptic inter-spike intervals over which synaptic strengthening and weakening occur. *A*_+_ and *A*_−_, which are both positive, determine the maximum amounts of synaptic modifications. Typical values of these parameters in our simulations were *A*_+_ = 0.013, *A*_−_ = 0.005, τ_+_ = 10 ms and τ_−_ = 9.5 ms.

Alternatively, we also considered the inverse STDP rule that has been observed, for example, in the sensory systems and cerebral cortex of fish (Bell et al., 1999; Zhigulin et al., 2003; Fino et al., 2005). In this case, the change in the synaptic strength due to learning at each time step is expressed as:

(7) $$\begin{array}{c} \Delta w_{ij}^{I}(\Delta \tau )=\{\begin{array}{c} \begin{array}{cc} -A_{+}\exp\;\left(-\Delta \tau /\tau_{+}\right) & \Delta \tau >0 \end{array}\\ \begin{array}{cc} A_{-}\exp\;\left(\Delta \tau /\tau_{-}\right) & \Delta \tau <0 \end{array} \end{array}. \end{array}$$

Typical values of these parameters in our simulations are *A*_+_ = 0.005, *A*_−_ = 0.013, τ_+_ = 9.5 ms and τ_−_ = 10 ms.

In this study, the matrix of synaptic strength is asymmetric. A change in the synaptic strength might result from remodeling of synapses in both presynaptic loci and postsynaptic terminals. Saturation of synaptic efficacy can occur after repeated potentiation, and previous experiments have shown that saturation of hippocampal LTP impairs spatial learning (Castro et al., 1989; Moser et al., 1998). Here, for simplicity, we did not introduce saturation for the synaptic efficacy since we studied the simplified case that the learning due to the external signal is turned off before the synaptic efficacy saturates. Nevertheless, saturation could be achieved by an appropriate choice of *A*_+_ and *A*_−_ as a function of the synaptic strength (*w*_*ij*_), which vanishes at the maximal *w*_*ij*_. We note that several other stabilization procedures can also terminate learning either when activity levels reach a certain threshold level or invoke a bound on synaptic weight strengths (Nass and Cooper, 1975; Linsker, 1986).

### Neural networks in a noisy environment

The dynamics of neural networks was studied by considering the HH model (Equations 1–4) in Section The Neuron Model and the synaptic plasticity rules (Equations 6, 7) in Section Synaptic Plasticity. In addition, in a noisy environment, electric noise could play an important role in the neuron dynamics. For example, in the experiment of hippocampal CA3 networks *ex vivo* by multi-neuron imaging technique (Takahashi et al., 2010), it was observed that neurons can synchronize in a noisy network. The main source of this noise is typically synaptic, resulting from the probabilistic release of synaptic vesicles and bombardment from the myriad of synapses made by other cells. Although neurons' firing frequency rarely exceeds 100 Hz, the combined synaptic activities of a neural network can produce fluctuations on a much faster time scale. Synaptic noise causes abrupt changes in the associated synaptic conductance each time a spike invades the pre-synaptic bouton. Stein's model describes its effect in the evolution of the membrane potential of a given neuron as trains of Dirac delta functions, which are summed up to become Gaussian white noise in the diffusion limit of synaptic input (Stein, 1967). Thus, in the presence of synaptic noise, the membrane potential of a network neuron can be solved using the Euler method as:

(8) $$\begin{array}{l} V_{i}(t+\Delta t)=V_{i}(t)\\ +\;\frac{\Delta t}{C_{m}}\left\{\begin{array}{l} I_{i}^{\text{syn}}(t)+g_{\text{Na}}m_{i}{\left(t\right)}^{3}h_{i}(t)[V_{\text{N}a}-V_{i}(t)]\\ +\;g_{\text{K}}n_{i}{\left(t\right)}^{4}[V_{K}-V_{i}(t)]+g_{\text{L}}[V_{\text{rest}}-V_{i}(t)]+I_{\text{noise}} \end{array}\right\}, \end{array}$$

where the Gaussian white noise (with the standard deviation *D*), *I*_noise_, was generated using the Box–Müller transform. The typical value of *D* in this study is 25 μA/cm^2^.

### Simulating coupled neural networks

For the simulation of coupled neural networks, we first built two independent neural layers (layers 1 and 2), each consisting of *M* = 50 excitatory HH neurons grafted on a square substrate of area 10^4^ (in the unit of soma area). In this study, for the positioning of neurons on each layer, we consider three different distributions, including a random distribution, a grid distribution, and a lognormal distribution. In the case of a lognormal distribution, the *x*- and *y*-coordinates of neurons were determined using the function min(100 × *f*_logn_ (*x*|0.5, 0.2), 100), where $f_{\text{logn}}\left(x|\mu ,\sigma \right)={\left(x\sigma \sqrt{2\pi }\right)}^{-1}\exp\left[-{\left(\ln\;x-\mu \right)}^{2}/2\sigma^{2}\right]$ is a lognormal function with the log mean μ and log standard deviation σ, and min(*a, b*) selects the smaller value from *a* and *b*. All neuron pairs have a distance longer than 1 unit length to avoid overlapping of neurons. Our motivation to use the lognormal distribution is to increase the population of neurons with high synaptic connectivity, which serve as hub neurons in a neural network and enhance network-wide synchronicity (Bonifazi et al., 2009).

For each neural layer, based on previous studies (Jia et al., 2004; Lin et al., 2011), we assume that the probability to form a synaptic connection between intra-layer neurons is inversely proportional to the distance between neurons, i.e., $p_{i,j}^{\text{intra}}=k/r_{i,j}$, where *r*_*i*__*j*_ is the distance between neurons *i* and *j*. Such a connection probability is valid if the process for a neuron to find another one is by a random search on a two-dimensional plane. The coefficient *k* in general depends on the level of activity in the network, such as local concentration of neurotrophins (Vicario-Abejón et al., 1998), and we used *k* = 0.005 in our simulations. The total number of intra-layer connections on a neural layer is denoted as *N*_c_(*N*_c1_ for layer 1, and *N*_c2_ for layer 2).

**Figure 1:**
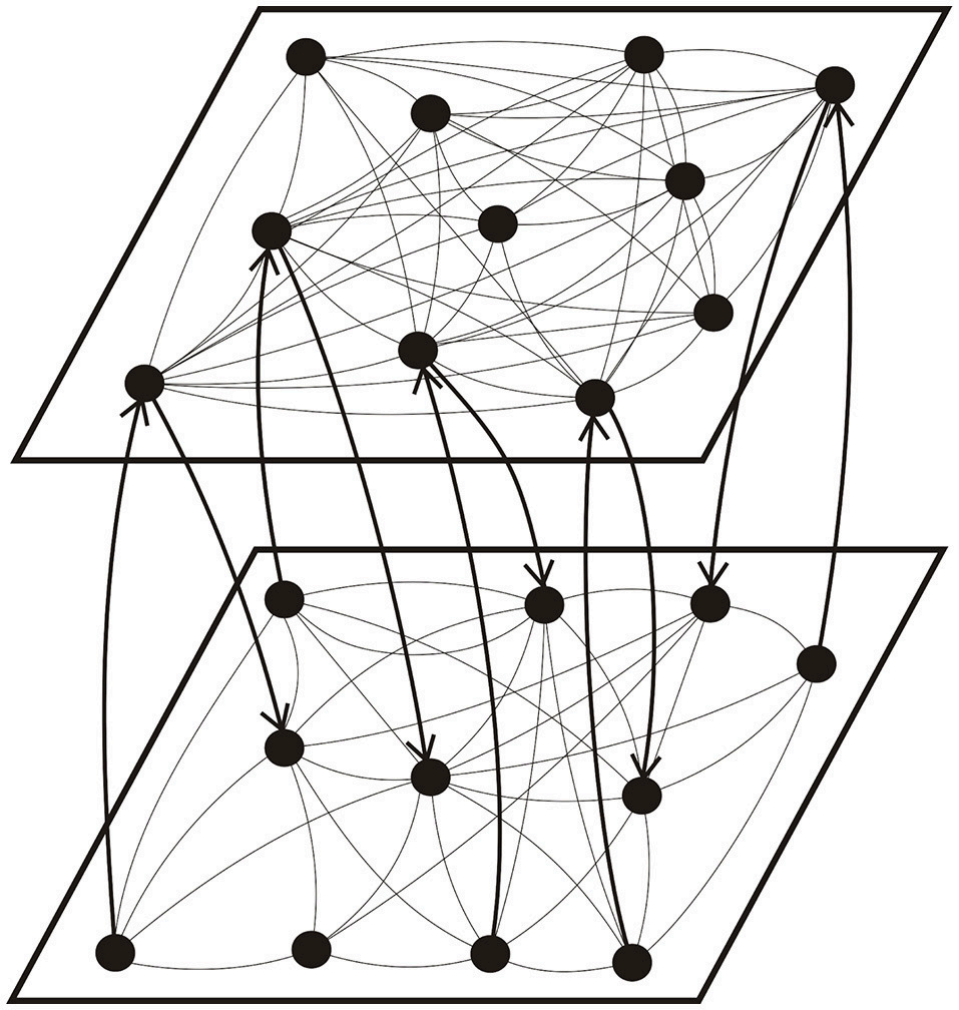
**Schematic illustration of the topology of a coupled network consisting of a high connectivity layer (top) and a low connectivity layer (bottom)**. The arrows show the direction of inter-layer synaptic connections.

For the coupling between two layers, as shown in Figure 1, we considered two types of inter-layer connections, including a random connection and a preferential connection. In the former case, the inter-layer synapses were established randomly between neurons with a fixed probability; while in the latter case, the probability to form an inter-layer synapse was proportional to the number of existing intra-layer connections of neurons *i* and *i*′. In other words, we assumed $p_{i,i^{\prime }}^{inter}=c_{i}c_{i^{\prime }}/{\left(M-1\right)}^{2}$, where *M* is the number of neurons per layer and *c*_*i*_ is the number of intra-layer connections of neuron *i* (neurons *i* and *i*′ belong to different layers). We differentiated between the connection from neuron *i* to neuron *j* and that from neuron *j* to neuron *i*, i.e., all intra- and inter-layer connections are directed. In our simulation, we assumed an equal number (*N*_i_) of inter-layer connections in either direction.

To simulate the dynamics of the coupled neural network, initially we set the membrane potential of neurons to have a Gaussian distribution with a zero average and a standard deviation of 5 mV and the synaptic strength between neurons to have a Gaussian distribution with an average of 0.025 and a standard deviation of 0.01. The simulation of network activities was divided into two parts, each containing a learning phase for 2 s and a recall phase for 3 s. The synaptic strength of each connection was updated according to learning rules (Equations 6 or 7) in the learning phase, but remained a constant value in the recall phase. In the first part of simulation, we studied the dynamics of two independent layers by varying the number of intra-layer synaptic connections (*N*_i_) and the maximum amounts of synaptic modification (*A*_+_ and *A*_−_), and obtained the phase diagram of each independent layer. In the second part of simulation, we investigated the interaction of these two layers by varying the number of inter-layer synaptic connections. In addition, the inter-layer connections were disconnected at the end of the recall phase in the second part of simulation, and we simulated the firing dynamics of the network for another 3 s (without learning) to investigate the stability of the induced synchronous firing state (SFS). We note that all parameters in our simulations were assumed to be their typical value unless otherwise specified. For each set of parameters, we simulated the network activities 30 times by using different seeds to measure the average value of related physical properties.

## Results and discussion

### Noise-driven synchronous firing of a developing neural network

The hypothesis that the brain computes information using neural synchronization has been supported by a mounting number of experimental evidence (Rodriguez et al., 1999; Fries, 2009). In this study, we first studied the noise-driven synchronous firing dynamics of a developing neural network (a single layer of 50 HH neurons) and derived its phase diagram. To begin with, there was no connection among neurons in the network. As the network developed with culturing time, intra-layer connections were established between neurons with the probability $p_{i,j}^{\text{intra}}$. As demonstrated in our previous study (Lin et al., 2011), as network connectivity increased, more and more neurons became active due to the presence of noise. Two neurons (*i* and *j*) were considered as synchronized in their firing pattern if both of them are active (fired at least once in the learning phase) and their time correlation (*TC*_*ij*_ ≡ *cov*(*V*_*i*_(*t*), *V*_*j*_(*t*))/σ_*i*_σ_*j*_, where *cov* means covariance and σ_*i*_ is the standard deviation of *V*_*i*_) is greater than 0.2 (Chao and Chen, 2005; Lin et al., 2011). The selected threshold value of time correlation for neural synchronization is usually not high since synchronous neuron firing only requires that the membrane potentials of synchronous neurons reach the threshold potential at the same time. By inspecting our simulation results, we selected 0.2 (which is not rigid) as the threshold value for indicating synchronous firing of neurons. Although there are some false positives in identifying synchronous neurons by using a low threshold value, they can be avoided by adding a constraint in the root mean square deviation of neurons' firing time to ensure that neurons' firing events are within a limited time span. Moreover, we defined an order parameter Ψ_s_ for the synchronization of neural activities as the number of synchronized neuron pairs divided by the total number of connections. In the present study, a network state is considered as a SFS if the time average of Ψ_s_ (〈Ψ_s_〉) in the recall phase is greater than 0.95, a transition state (TS) if 0.4 ≤ 〈Ψ_s_〉 ≤ 0.95, and a background activity state (BAS) if 〈Ψ_s_〉 < 0.4.

**Figure 2:**
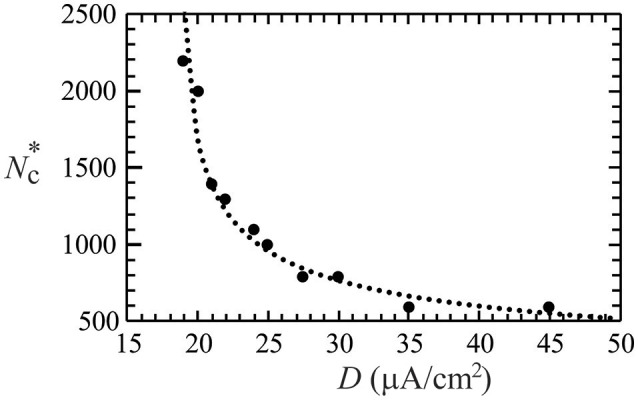
The threshold value ($N_{c}^{*}$) of network connections to induce synchronous firing as a function of the standard deviation (***D***) of a Gaussian white noise. The dotted curve is a fit of the data points, which suggests no synchronization for *D* ≤ 18.5 μA/cm^2^. Data were calculated by averaging 30 realizations and using the following set of parameters: *A*_+_ = 0.013 and *M* = 50.

In non-linear dynamical systems, it is known that non-trivial effects of noise could lead to synchronization of the system (Neiman et al., 1998; Zhou and Kurths, 2003). Such noise-driven synchronization is a topic of relevance to neuroscience, and has been experimentally observed in animal neocortical neurons (Mainen and Sejnowski, 1995). By varying the degree of network development (i.e., the degree of network connections, *N*_c_), here we investigate the effect of a Gaussian noise (of standard deviation *D*) on the synchronous firing of a neural network with STDP learning rule, which consists of 50 neurons randomly distributed on a square substrate. In our simulations, no synchronous firing was observed for networks at early developmental stages (small *N*_c_). As the network further developed, synchronous firing was observed for *N*_c_ greater than a threshold value ($N_{\text{c}}^{*}$). The value of $N_{\text{c}}^{*}$ was found to increase as *D* decreases, and no synchronous firing was observed for *D* = 18.5. Figure 2 delineates the dependence of $N_{\text{c}}^{*}$ on the standard deviation (*D*) of *I*_noise_, in which the dotted curve is a fit of data points using $N_{\text{c}}^{*}$ = 2000/(*D* − 18.5)^0.4^. Our results suggest that noise of a large *D* value can effectively enhance the coherence of the spike trains in a network of coupled neurons.

**Figure 3:**
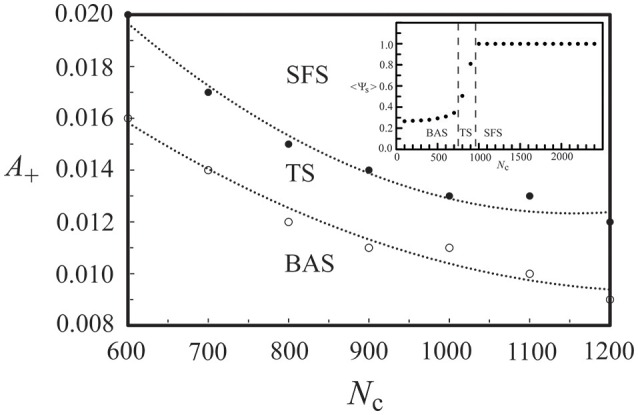
**Phase diagram of a developing neural network, which consists of a background activity state (BAS), a transition state (TS), and a synchronous firing state (SFS)**. The inset shows the synchronization order parameter <Ψ_s_> at various values of network connectivity. Data were calculated by averaging 30 realizations and using the following set of parameters: *A*_+_ = 0.013, *D* = 25 μA/cm^2^, and *M* = 50.

**Figure 4:**
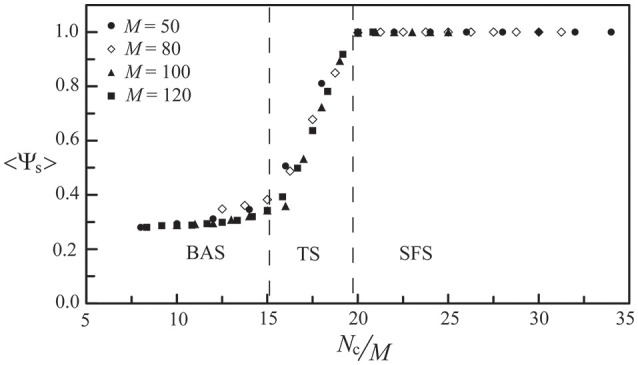
**The synchronization order parameter as a function of the average number of connections per neuron (***N***_***c***_/***M***) for ***M*** = 50, 80, 100, and 120**. Data were calculated by averaging 30 realizations and using the following set of parameters: *A*_+_ = 0.013 and *D* = 25 μA/cm^2^.

For the above neural network, its phase diagram is shown in Figure 3 for *A*_−_ = 0.005 and *D* = 25 μA/cm^2^. The data points in Figure 3 were obtained from calculating the synchronization order parameter (as shown in the inset) and the dotted lines were fitted curves for these data points. In general, for a neural network with the STDP rule, SFS is usually found in the region of large *A*_+_ and large *N*_c_, while BAS exists in the small *A*_+_ and small *N*_c_ region of the phase diagram. This observation is consistent with the results in our previous study (Lin et al., 2011). Recent experiments on the synaptic plasticity in mouse barrel cortex have shown that the learning efficacy declines and disappears with age (Banerjee et al., 2009; Kaczorowski and Disterhoft, 2009). Therefore, the BAS network might be associated with neural systems that are aged (small *A*_+_) or undeveloped (small *N*_c_). The inset of Figure 3 shows the value of 〈Ψ_s_〉 as a function of *N*_c_ (intra-layer connection), which increases sharply for *N*_c_ > 700 and saturates around *N*_c_ = 1000. Such a narrow TS region (about a range of 300 in *N*_c_) sandwiched by BAS and SFS is also seen for other sets of parameters, as indicated by Figure 3. To further discuss the finite size effect on the transition from BAS to SFS, in Figure 4, we calculated the average synchronization order parameter as a function of the average number of connections per neuron (*N*_c_/*M*) for different network sizes (*M* = 50, 80, 100, and 120). In general, data points in Figure 4 collapse into a single curve, suggesting a small finite size effect on the BAS-SFS transition. However, a slightly sharper transition is observed for networks of larger sizes. In our model, SFS is observed if the average number of connections per neuron (*N*_c_/*M*) is greater than 20 for networks of size between 50 and 120 neurons.

### Interaction of two neural layers

**Figure 5:**
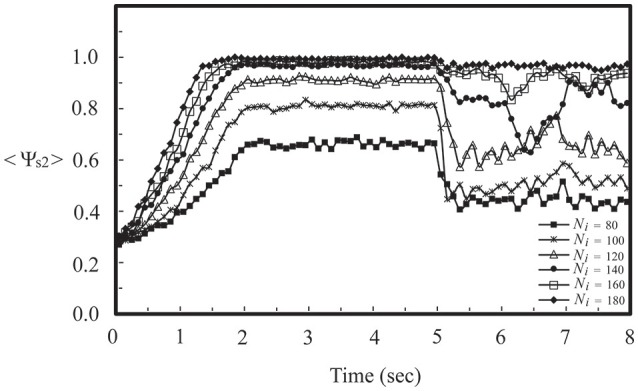
**Synchronization order parameter of a BAS layer (layer 2, ***N***_**c2**_ = 1000 and ***A***_**+**_ = 0.010) as a function of simulation time for various values of ***N***_**i**_ after its momentary coupling to a SFS layer (layer 1, ***N***_**c1**_ = 1000 and ***A***_**+**_ = 0.013)**. The time average <Ψ_s2_> was calculated by the average of 30 simulations with different seeds.

**Figure 6:**
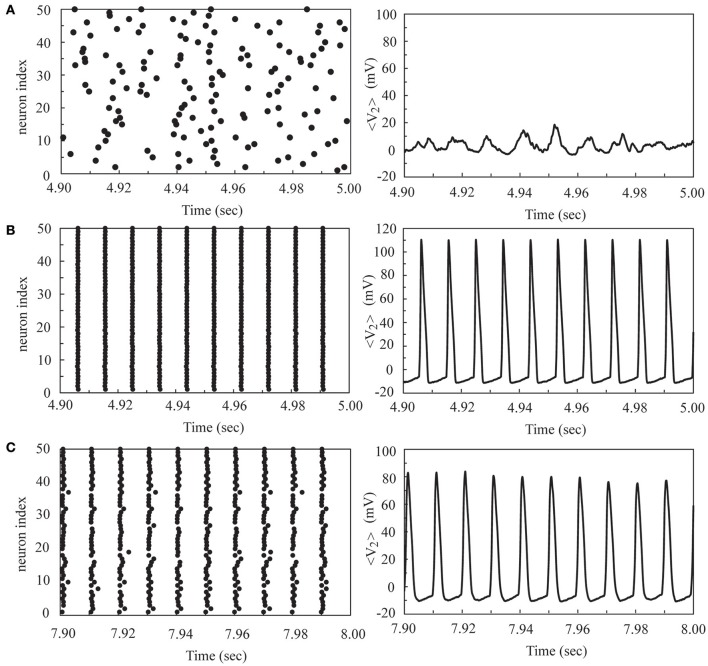
**The time series of neuron firing and the average membrane potential of layer 2 before coupling (A)**, after coupling **(B)**, and after disconnecting the coupling **(C)** to a SFS layer. Here we used the following set of parameters: *N*_c2_ = 1000 and *A*_+_ = 0.010 for layer 2, *N*_c1_ = 1000 and *A*_+_ = 0.013 for layer 1, and *N*_i_ = 180.

To further our investigation, we considered the firing dynamics of a network consisting of two weakly coupled neural layers. Each layer is composed of 50 neurons randomly positioned on the substrate. There are *N*_c1_ intra-layer connections for layer 1 and *N*_c2_ intra-layer connections for layer 2. Depending on the number of intra-layer connections, these two layers could be in a SFS, a TS, or a BAS before we coupled them together, as described in Section Noise-Driven Synchronous Firing of a Developing Neural Network. Here we first studied the interaction of a SFS layer (layer 1, *N*_c1_ = 1000 and *A*_+_ = 0.013) and a BAS layer (layer 2, *N*_c2_ = 1000 and *A*_+_ = 0.010), which were randomly connected with *N*_i_ inter-layer connections. The inter-layer connections were introduced at *t* = 0 and the learning phase lasted for 2 s, followed by a recall phase for 3 s. For each *N*_i_, 30 realizations were simulated with different seeds to calculate the synchronization order parameter of layer 2. For each realization, the inter-layer connections were varied, while the intra-layer connections were fixed. To study the robustness of the induced synchronization of the BAS layer by a small inter-layer coupling, we then removed all inter-layer connections from this network, and simulated the activities of the network for 3 s (in a recall phase). The average synchronization order parameter of layer 2 is displayed in Figure 5 for various numbers of inter-layer connections, *N*_i_. For a coupled network with *N*_i_ ≤ 120, layer 2 did not reach a SFS after 2 s learning, and the order parameter dropped significantly (less than 0.6) after the removal of all inter-layer connections. For *N*_i_ = 140, layer 2 has reached a SFS after 2 s learning, but the order parameter fluctuated largely after removing inter-layer connections. For *N*_i_ > 160, after 2 s learning, layer 2 reached a SFS and fired synchronously even after the inter-layer coupling was disconnected. It is known that both SFS and BAS are important states for neural computations. Our simulations confirm that SFS occurs for networks with large connectivity or large learning efficiency. These SFS networks can also be desynchronized by various control mechanisms, such as the inhibition control using GABAergic neurons (Treviño, 2016). On the other hand, for those network structures that generate BAS, there is no known efficient mechanism to induce synchronization in these networks without changing the network connectivity or inter-neuron interactions. Therefore, we consider region for SFS in the phase diagram as a better substrate of neural computation than that for BAS. This investigation demonstrates a possible mechanism for the repair or an enhanced learning of a BAS neural layer (possibly resulting from aging, immaturity, or external damage) by coupling it with a SFS layer. Furthermore, we investigated the induced synchronization of a BAS layer by its coupling to a SFS layer for the case of *N*_i_ ≥ 180 in Figure 5. The BAS layer was considered as an aged neural layer, which had a learning efficacy (*A*_+_ = 0.010) smaller than that (*A*_+_ = 0.013) of a normal SFS layer with the same degree of intra-layer connectivity (*N*_c1_ = *N*_c2_ = 1000). In Figure 6, we showed the time series of neuron firing and the average neuronal membrane potential (<*V*_2_>) of layer 2 before coupling (a), after coupling (b), and after disconnecting the coupling (c), all of which were recorded in the recall phase. Before coupling, layer 2 showed a random firing pattern and its average membrane potential was usually smaller than the firing threshold (≅ 6.9 mV). After coupling, layer 2 was driven to fire synchronously and its average membrane potential showed a periodic spiking pattern. After the removal of all inter-layer synaptic connections, this periodic spiking persisted for a long time (as shown in Figure 5). We note that, as described earlier for Figure 5, there is no learning phase after decoupling the inter-layer connections.

**Figure 7:**
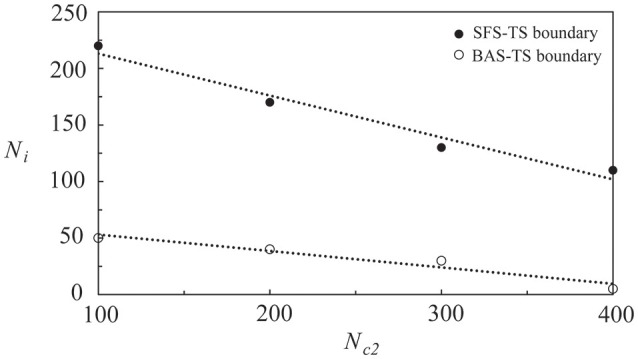
**Phase diagram of layer 2 in a network of two coupled neural layers by varying ***N***_**i**_ and ***N***_**c2**_**. The phase diagram was plotted based on <Ψ_s_>, which was calculated by the average of 30 simulations with different seeds. Here we used the following set of parameters: *N*_c1_ = 1000, and *A*_+_ = 0.013 for both layers.

In Figure 7, we showed the phase diagram of layer 2 in a coupled network of two layers, in which *N*_c1_ = 1000 and *N*_c2_ is variable. Before coupling, layer 1 was in the SFS, layer 2 was in the BAS, and both layers had a learning efficacy *A*_+_ = 0.013. By varying the number of inter-layer connections, *N*_i_, layer 2 was driven to become a SFS layer after learning. The dotted lines showed phase boundaries in the phase diagram that were linearly fitted using the data points in Figure 7. For *N*_c2_ = 100, the value of *N*_i_ is about 50 for a transition from a BAS layer to a TS layer, and is about 220 for a transition from a TS layer to a SFS layer. These critical values of *N*_i_ decrease linearly with *N*_c2_. It is also observed that the range of *N*_i_ for the existence of a TS layer becomes narrower at larger *N*_c2_.

**Figure 8:**
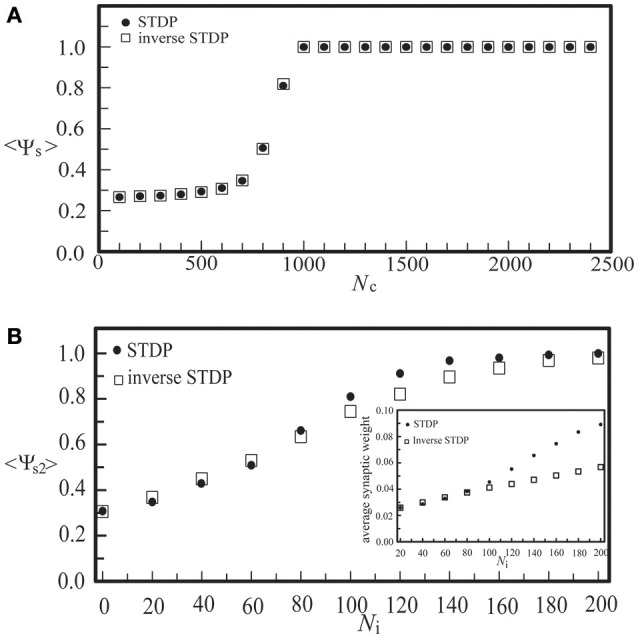
**The synchronization order parameter of a BAS layer as a function of ***N***_***c***_ in a single-layer noisy network (A), or as a function of ***N***_**i**_ in a coupled network consisting of a BAS layer (***N***_**c2**_ = 300) and a SFS layer (***N***_**c1**_ = 1000) (B)**. Both the STDP (*A*_+_ = 0.013) and the inverse STDP (*A*_−_ = 0.013) learning rules were considered. The synchronization order parameter was calculated by its average of 30 simulations with different seeds.

To compare the learning effects of the STDP and the inverse STDP in inducing network synchronization, as shown in Figure 8, we calculated the synchronization order parameter of layer 2 using these two learning rules in a single layer network (a) and a network consisting of two coupled neural layers (b). For the case of a single layer network with various values of *N*_c_, as shown in Figure 8A, we found no difference in the effects of the STDP or the inverse STDP rules since there were roughly equal amount of firing events with positive Δτ or negative Δτ (defined in Equations 6, 7), and thus their contribution to the change of average network synaptic weight was similar with these two learning rules. For the case of two coupled layers in which layer 1 was a SFS layer and layer 2 was a BAS layer, there were more synaptic currents from the direction of layer 1 to layer 2 than the opposite direction. Such an asymmetry led to larger enhancements in the average inter-layer synaptic weight with the STDP learning rule, as shown in the inset of Figure 8B. For *N*_i_ > 200, the synchronization order parameter of layer 2 saturated with either learning rule. Nevertheless, there still was a large difference in the average inter-layer synaptic weight of the network with these two learning rules.

**Figure 9:**
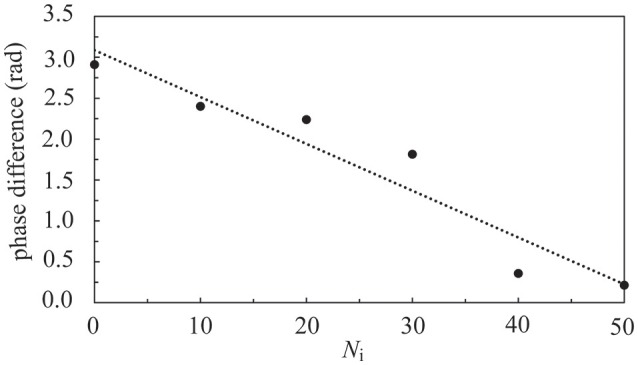
**Phase difference of two coupled SFS layers as a function of ***N***_**i**_**. Before coupling, these two layers fired at the same frequency but with a phase difference of 2.9 radians. Data were calculated by averaging 5 realizations and using the following set of parameters: *N*_c1_ = *N*_c2_ = 1000 and *A*_+_ = 0.013 for both layers.

In Figure 9, we investigated the interaction of two neural layers (*N*_c1_ = *N*_c2_ = 1000, and *A*_+_ = 0.013 for both layers) having the same frequency but different phases in their synchronous firing pattern. Before the coupling, both layers were in the SFS, and the synchronous firing pattern of layer 1 was lagging behind that of layer 2 by 0.005 s (or a phase difference of 2.9 radians). The inter-layer connections were introduced at *t* = 0. The learning phase lasted for 2 s, which was followed by a recall phase for 3 s. After introducing the inter-layer coupling, in the recall phase, we observed a phase shift of layer 1 due to the synaptic currents from layer 2 as shown in Figure 9. We note that the data points in Figure 9 were obtained from 5 realizations with the same initial phase difference between the two layers, and the dotted line is a linear fit of data points. As *N*_i_ increases, the phase difference between the two layers diminishes. At *N*_i_ > 50, the two layers almost fired simultaneously.

### Designs of coupled neural networks

**Figure 10:**
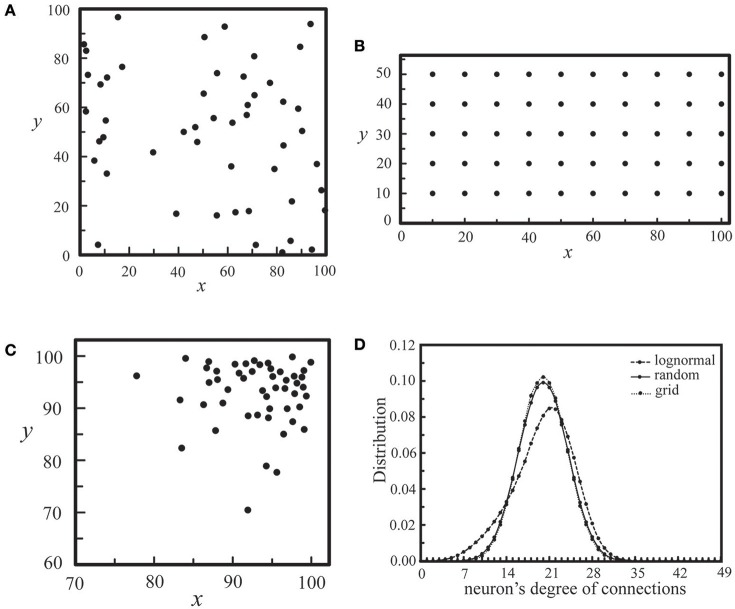
**Three types of neuron positioning on a layer, including a random distribution (A), a grid distribution (B), and a lognormal distribution (C)**. For all three cases, *N*_*c*_ = 1000. In **(D)**, we showed the distribution of neurons' degree of connections for the three types of neuron positioning in **(A–C)**, each of which was calculated by averaging an ensemble of 1000 seeds.

As neural synchronization plays an important role in brain's information processing, we would like to explore some possibilities in the positioning of neurons on each layer and in the mechanism of inter-layer connections by designing coupled neural networks, and find out designs that may strengthen their synchronization activities. The realization of these designs might rely on experimental controls of neurotrophins (Vicario-Abejón et al., 1998) as well as neuron positioning related genes, such as the reeler gene and the mouse disabled1 gene (Stanfield and Cowan, 1979; Howell et al., 1997). Similar to those networks discussed in Section Interaction of Two Neural Layers, the coupled networks under our investigation here consist of two coupled neural layers, each of which is composed of 50 neurons. For each layer, as shown in Figure 10, we considered three types of neuron positioning, including a random distribution (a), a grid distribution (b), and a log-normal distribution (c). For these three types of neuron positioning, the degree of intra-layer synaptic connections of each neuron was calculated using the connection rule described in Section Simulating Coupled Neural Networks and averaged over 1000 seeds, as shown in Figure 10D. The total number of intra-layer connections is 1000 for all three cases. It is seen that the distribution of intra-layer connections is almost the same for the cases of (a) and (b), and we expect little difference in their synchronization behavior. On the other hand, for the lognormal positioning of neurons, there is a larger population of neurons which have a large intra-layer connectivity, and we expected to see some enhancement in the synchronization behavior of the network.

**Figure 11:**
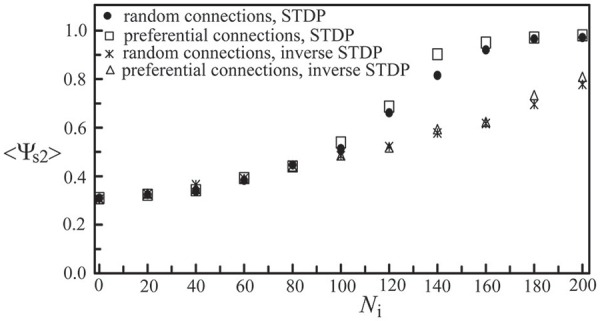
**Synchronization order parameter of layer 2 as a function of ***N***_**i**_**. Four scenarios of different inter-layer connection mechanisms and learning rules were displayed, including a network with the random connection and the STDP learning, one with the preferential connection and the STDP, one with the random connection and the inverse STDP, and the other with the preferential connection and the inverse STDP. Here we used the following set of parameters: *N*_c1_ = 1000, *N*_c2_ = 300, and *A*_+_ = 0.013 for both layers. The time average <Ψ_s2_> was calculated by the average of 30 simulations with different seeds.

**Figure 12:**
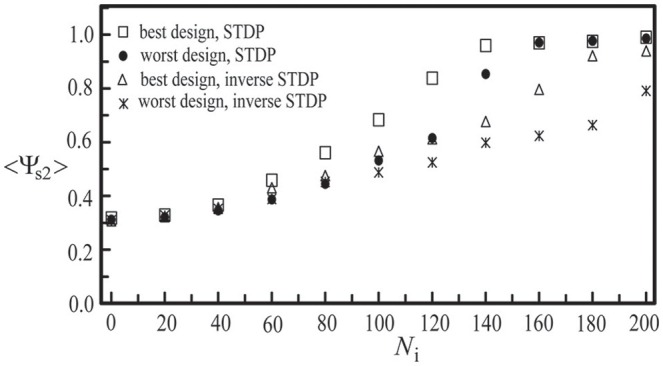
**Synchronization order parameter of layer 2 as a function of ***N***_**i**_ to demonstrate our best and worst designs of two coupled neural layers in synchronization enhancement**. Both the STDP and the inverse STDP learning rules were considered. Here we used the following set of parameters: *N*_c1_ = 1000, *N*_c2_ = 300, and *A*_+_ = 0.013 for both layers. The time average <Ψ_s2_> was calculated by the average of 30 simulations with different seeds.

Next, we studied the network synchronization behavior by considering two different mechanisms of inter-layer connections as described in Section Simulating Coupled Neural Networks: (a) the random connection and (b) the preferential connection. For the coupled network (*N*_c1_ = 1000, *N*_c2_ = 300) with a random positioning of neurons and *N*_i_ inter-layer connections, in Figure 11, we compared the synchronization order parameter of layer 2 for these two connection mechanisms. After the two layers were momentarily coupled, it was found that the value of <Ψ_s2_> increased due to the interaction between the two layers. For 100 < *N*_i_ < 200, the enhancement in the synchronization order parameter of layer 2 was larger in the case of the preferential connection. Furthermore, we considered six designs of coupled neural networks (three positioning schemes × two connection schemes) and investigated the synchronization enhancement of a BAS layer by its momentary coupling to a SFS layer. As demonstrated in Figure 12, the largest enhancement was observed for the scenario with a lognormal positioning of neurons and the preferential inter-layer connections, and the smallest enhancement was observed for the scenario with a grid positioning of neurons and the random inter-layer connections. These results are valid for networks with the STDP or the inverse STDP learning rules.

## Conclusion

In conclusion, we have applied a HH model of neurons to study the synchronization behavior of a noisy neural network (a single layer), and obtained its phase diagram by varying the network connectivity (*N*_c_) and the learning efficacy (*A*_+_). The phase diagram shows the existence of three network states (SFS, TS, and BAS) and boundaries between these states. The BAS could occur in regions of small *A*_+_ or small *N*_c_. Based on this phase diagram, we have investigated the interaction of two coupled neural layers, which are in different states (SFS for layer 1 and BAS for layer 2), or in the same SFS state but with a phase difference in their firing patterns. For a coupled neural network consisting of a SFS layer and a BAS layer with 140 < *N*_i_ < 180, the BAS layer fires synchronously after 2 s learning, but the synchronization is unstable after disconnecting inter-layer connections. For *N*_i_ ≥ 180, the induced synchronization in the BAS layer is stable even after the inter-layer connections are removed. Such an induced synchronization of BAS layers could be considered as a repair mechanism of neural networks. The phase diagram of coupled neural networks has also been derived. We have further considered the effect of this repair mechanism by varying neuron positioning on each layer (random, grid, or lognormal distributions) and the probability of inter-layer connections (random or preferential connections). We have concluded that, for both the STDP and the inverse STDP, a combination of the lognormal neuron positioning and the preferential inter-layer connections has the largest enhancement in network synchronization in these designs.

## Author contributions

AY is responsible for writing the main code for the study of interacting neural networks and conducting numerical studies for various parameter values. TM helped to code several algorithms, studied the noise effects, and provided helpful discussions. CC supervised the study and prepared the manuscript.

### Conflict of interest statement

The authors declare that the research was conducted in the absence of any commercial or financial relationships that could be construed as a potential conflict of interest.
